# Cell Killing Mechanisms and Impact on Gene Expression by Gemcitabine and ^212^Pb-Trastuzumab Treatment in a Disseminated i.p. Tumor Model

**DOI:** 10.1371/journal.pone.0159904

**Published:** 2016-07-28

**Authors:** Kwon Joong Yong, Diane E. Milenic, Kwamena E. Baidoo, Martin W. Brechbiel

**Affiliations:** Radioimmune & Inorganic Chemistry Section, Radiation Oncology Branch, National Cancer Institute, National Institutes of Health, Bethesda MD, United States of America; Roswell Park Cancer Institute, UNITED STATES

## Abstract

In pre-clinical studies, combination therapy with gemcitabine and targeted radioimmunotherapy (RIT) using ^212^Pb-trastuzumab showed tremendous therapeutic potential in the LS-174T tumor xenograft model of disseminated intraperitoneal disease. To better understand the underlying molecular basis for the observed cell killing efficacy, gene expression profiling was performed after a 24 h exposure to ^212^Pb-trastuzumab upon gemcitabine (Gem) pre-treatment in this model. DNA damage response genes in tumors were quantified using a real time quantitative PCR array (qRT-PCR array) covering 84 genes. The combination of Gem with α-radiation resulted in the differential expression of apoptotic genes (*BRCA1*, *CIDEA*, *GADD45α*, *GADD45γ*, *IP6K3*, *PCBP4*, *RAD21*, and *p73*), cell cycle regulatory genes (*BRCA1*, *CHK1*, *CHK2*, *FANCG*, *GADD45α*, *GTSE1*, *PCBP4*, *MAP2K6*, *NBN*, *PCBP4*, and *SESN1*), and damaged DNA binding and repair genes (*BRCA1*, *BTG2*, *DMC1*, *ERCC1*, *EXO1*, *FANCG*, *FEN1*, *MSH2*, *MSH3*, *NBN*, *NTHL1*, *OGG1*, *PRKDC*, *RAD18*, *RAD21*, *RAD51B*, *SEMA4G*, *p73*, *UNG*, *XPC*, and *XRCC2*). Of these genes, the expression of *CHK1*, *GTSE1*, *EXO1*, *FANCG*, *RAD18*, *UNG* and *XRCC2* were specific to Gem/^212^Pb-trastuzumab administration. In addition, the present study demonstrates that increased stressful growth arrest conditions induced by Gem/^212^Pb-trastuzumab could suppress cell proliferation possibly by up-regulating genes involved in apoptosis such as *p73*, by down-regulating genes involved in cell cycle check point such as *CHK1*, and in damaged DNA repair such as *RAD51* paralogs. These events may be mediated by genes such as *BRCA1/MSH2*, a member of BARC (BRCA-associated genome surveillance complex). The data suggest that up-regulation of genes involved in apoptosis, perturbation of checkpoint genes, and a failure to correctly perform HR-mediated DSB repair and mismatch-mediated SSB repair may correlate with the previously observed inability to maintain the G2/M arrest, leading to cell death.

## Introduction

Combination therapy with radiation and chemotherapeutics, a commonly used regimen for the treatment of cancer, highly improves therapeutic response. Due to a high linear transfer (LET) and a short range in tissue, alpha (α)-particles induce clusters of DNA strand breaks, leading to cell death [1–5]. Thus, high-LET radiation with less damage to surrounding normal tissue is more specific and effective in cell killing than low-LET radiation such as β^−^-particles [6–8]. Several α-emitting radionuclides have been successfully used in radioimmunotherapy (RIT) for targeted therapy of cancer [9–12]. When applied as a monotherapy or in combination with chemotherapeutics, radioimmunotherapies with ^212^Pb have shown the high therapeutic efficacy of this isotope in targeted α-particle therapy for disseminated peritoneal diseases [9, 13–16].

Gemcitabine (Gem), a well-defined FDA approved chemotherapeutic, is a nucleoside analogue widely used as the first-line chemotherapy against cancer. It has demonstrated the therapeutic feasibility as a single modality against tumors [17–20]. As such, Gem in conjunction with ^212^Pb-trastuzumab was evaluated as one of chemotherapeutics, the combination of which was reported to significantly enhance therapeutic response [15, 16].

In response to DNA breaks, catastrophic cellular injury that causes failure in maintaining the genetic integrity, leading to cell death results via a variety of mechanisms such as apoptosis, autophagy, necrosis, and mitotic catastrophe. Radiation-induced complex signaling pathways and alterations in gene expression may provide valuable information to identify potential biomarkers of human response to radiation [21]. Tissue response and associated gene modulations have, however, not been clearly defined following exposure of tumors to α-particle RIT unlike the many possibilities that are described for chemotherapy. Recently, gene expression profiles in different biological systems have been identified following exposure to high-LET radiation such as α-particles. In comparison with ^60^Co in human fibroblasts, biological processes such as mitosis, spindle assembly checkpoint, and apoptotic chromosome condensation were uniquely modified after exposure to α-particle radiation (^211^At-labeled trastuzumab), suggesting α-particle radiation clearly influenced tumor protein p53-activated and repressed genes [22]. Pathway analysis associated with differentially modulated genes in human lung epithelial cells exposed to α-particle radiation (^222^Rn) suggested that α-particle radiation inhibits DNA synthesis and subsequent mitosis, and caused cell cycle arrest via p53 signaling. Seidl and colleagues demonstrated that cell killing by α-particle radiation (^213^Bi-d9MAb) in human gastric cancer cells (HSC45-M2) was evident in the formation of micronuclei and severe chromosomal aberrations. In gene expression profiling for the whole genome, up-regulated genes (*COL4A2, NEDD9,* and *C3*) and down-regulated genes (*WWP2, RFX3, HIST4H4,* and *JADE1*) were unique, which were not related to any biological processes [23–25].

In response to α-particle RIT combined with an established chemotherapeutic agent such as Gem, application of gene expression profiling may reveal potential clinical targets by providing novel information for further biomedical and clinical research. For this purpose, the gene modulation in tumors that received Gem combined with specifically targeted α-particle RIT (^212^Pb-trastuzumab) in the LS-174T i.p. xenograft model is described using a real time quantitative PCR (qRT-PCR) array to investigate key biological processes such as apoptosis, cell cycle arrest, and DNA repair with regard to gene expression.

## Materials and Methods

### Cell line

All of the *in vivo* studies were conducted using the human colon carcinoma cell line (LS-174T; provided by Dr. J. Greiner, NCI, Bethesda, MD) grown in supplemented Dulbecco’s Modified Eagle’s Medium (DMEM) as previously described by Tom BH et al [26] with all media and supplements being purchased from Lonza (Walkersville, MD) unless otherwise indicated. The cell line was screened for mycoplasma and other pathogens before *in vivo* use according to National Cancer Institute (NCI) Laboratory Animal Sciences Program policy without any further cell line authentication.

### Chelate synthesis, mAb conjugation, and radiolabeling

The synthesis, characterization, and purification of the bifunctional ligand TCMC have been previously described [27]. Conjugation of trastuzumab (Herceptin^®^; Genentech, South San Francisco, CA) was conducted with TCMC by established methods using a 10-fold molar excess of ligand to mAb. A 10 mCi ^224^Ra/^212^Pb generator (AlphaMed, Lakewood, NJ) was washed with 2 M HCl to remove any impurities and any unbound ^224^Ra. ^212^Pb was eluted from the generator with 1 M HCl and dried. The residue dissolved in 0.1 M HCl was used for radiolabeling of mAb. The radiolabeled mAb was purified using a desalting column (GE Healthcare, Piscataway, NJ) with PBS. Purified polyclonal IgG (HuIgG) fraction was similarly conjugated with TCMC and radiolabeled with ^212^Pb as described above, providing a non-specific control antibody for the experiments.

### Tumor model, treatment and tumor harvesting

All animal protocols were approved by the National Cancer Institute (NCI) Animal Care and Use Committee for all experiments. To provide ample space to mice, five female mice were housed per autoclaved cage at the NCI vivarium with bedding and nesting materials provided in each cage. The mice were also provided with sterile mouse chow and drinking water. The mouse chow and water were stored in clean, dedicated areas of the vivarium. All equipment and supplies entering the facilities were sterilized for animal health and well-being. Monitoring animals for health problems were performed on a daily basis. Any animal experiencing rapid weight loss, debilitating diarrhea, rough hair coat, hunched posture, labored breathing, lethargy, persistent recumbence, jaundice, anemia, significantly abnormal neurological signs, bleeding from any orifice, self-induced trauma, impaired mobility, or difficulty eating or drinking were immediately euthanized. Mice bearing i.p. xenografts may manifest additional clinical signs of disease progression such as sizeable abdominal distention, ascites or generalized subcutaneous edema and were euthanized. Mice experiencing significant weight loss or gain (10%, determined by weekly weighings) were also determined to reach the experimental/humane endpoints and were euthanized. Euthanasia was performed by removing the animal(s) from the home cage, and placing it in a chamber with a specialized euthanasia lid attached to a CO_2_ line. CO_2_ was allowed to flood the chamber at a rate of 2 L/min. When breathing ceased for all mice, the mice were removed from the chamber.

*In vivo* studies were performed with 19–21 g female athymic mice (NCI-Frederick). Athymic mice were injected i.p. with 1 x 10^8^ LS-174T cells in 1 mL of DMEM as previously reported [27]. The ^212^Pb-TCMC-trastuzumab (10 μCi) was administrated to the mice (n = 10–15) 3 days post-implantation of tumor in 0.5 mL PBS. HuIgG labeled with ^212^Pb served as the non-specific control. The α-radiation was administrated 3 d after tumor implantation. Gemcitabine (Eli Lilly, Indianapolis, IN), obtained through the NIH Division of Veterinary Resources Pharmacy, was prepared for injection at 1 mg/ 0.5 mL phosphate-buffered saline (PBS) and given by i.p. injection to the mice 2 d after injection of the LS-174T cells. This treatment group was compared with sets of tumor bearing mice that received gemcitabine alone, Gem/^212^Pb-HuIgG, or no treatment. Mice were euthanized 24 h after receiving the Gem/^212^Pb-RIT, the tumors harvested and stored at -80°C until use.

### RNA purification

To produce high quality RNA from tumor tissues (^212^Pb-trastuzumab treated or non-specific controls), total RNA isolation from tissue was performed using the RNeasy mini kit (Qiagen, Santa Clarita, CA) in accordance with the manufacturer’s instructions. Quantity and quality of isolated total RNA were assessed using Nano-drop spectrophotometer (Thermo Scientific, Wilmington, DE) using OD_260_ for calculation of concentration. Only that total RNA with an A260/A280 ratio > 1.9 and without detectable contamination of DNA (PCR) was employed in the gene expression array (qRT-PCR array).

### Human DNA damage PCR array

The human DNA damage PCR array (SABiosciences, Frederick, MD) profiles expression of 84 genes involved in apoptosis, cell cycle and damaged DNA binding and repair (S1 Table). cDNA was prepared from RNA using the First strand cDNA synthesis Kit (SABiosciences, Fredrick, MD). Comparison of the relative expression of 84 genes was characterized (RT^2^ real-time SYBR Green/Rox PCR master mix, SABiosciences) in 96 well microtiter plates on a 7500 real time PCR system (Applied Biosystems, Rockville, MD). Data was analyzed using the RT^2^ profiler PCR Array Data Analysis v3.5 software (Qiagen). The fold change in gene expression was calculated using the equation 2(-ΔΔC_T_). If the fold change was greater than 1, the result was considered as an up-regulation. For down-regulated (less than 1-fold change) genes the value was reported as the negative inverse.

### Chromatin immunoprecipitation

The chromatin immunoprecipitation (ChIP) assay kit (Upstate Biotechnology, Billerica, MA) was performed in accordance with the manufacturer’s instructions with minor adjustments. In brief, lysates from tumor tissues were prepared and aliquoted. Chromatin was immunoprecipitated with 10 μL (1:100) of antibody for E2F1 (Upstate Biotechnology). Antibody was incubated overnight with chromatin on a rotator at 4°C; the resulting DNA-protein complexes were isolated using protein G agarose magnetic beads. The samples were subjected to 65°C for 5 h, the DNA extracted, and dissolved in the elution reagent. The PCR-amplified DNAs using *CHK1*, *MSH2 and p73* promoter specific primers (Applied Biosystems) were analyzed by electrophoresis using 2% agarose gels.

### Immunoblot analysis

Total protein isolates using tissue protein extraction reagent (T-PER) (Thermo Scientific, Asheville, NC) containing protease inhibitors (Roche, Indianapolis, IN) were prepared for immunoblot analysis. Equivalent amounts of protein extracts were resolved on a 4–20% tris-glycine gel electrophoresis system and transferred to a nitrocellulose membrane. For immmunodetection, antibodies against RAD51B and XRCC2 (Abcam, Cambridge, MA) were used at a dilution of 1:1000 in PBS containing 5% BSA and 0.05% Tween-20 for 1 h. Horseradish peroxidase conjugated rabbit secondary antibodies were used at a 1:5000 dilution prepared in PBS with 3% non-fat dry milk. The immunoblots were developed using the enhanced chemoluminescent detection kit (GE Healthcare, Pascataway, NJ).

### Statistics

A minimum of at least three independent experiments were conducted for each treatment described. Statistical differences between the groups were determined using Student *t* test. For multiple comparisons, the ANOVA was performed. Statistically significant difference between datasets was determined at *p*-value < 0.05.

## Results

### Gemcitabine may potentiate α-radiation-induced cell killing by regulation of genes involved in apoptosis

Significantly up- or down-regulated genes 24 h after exposure of tumors to ^212^Pb-RIT in combination with Gem (n = 3) were identified through application of a 2-fold change threshold using qRT-PCR array as compared to the untreated group as a control. Thirteen of the 84 genes of DNA damage signaling pathway investigated in this study are associated with the regulation of the apoptotic process. Of these affected genes, six genes (*CIDEA*, *GADD45α*, *GADD45γ*, *IP6K3*, *PCBP4*, and *p73*) were up-regulated and two genes (*BRCA1* and *Rad21*) were down-regulated to varying degrees among the various treatment groups (Table 1). The expression of *p73* of the up-regulated genes appeared to exhibit the greatest impact from Gem/^212^Pb-trastuzumab (8.6-fold increase, *p* < 0.0021) and Gem/^212^Pb-HuIgG (10.1-fold increase, *p* < 0.0005) treatment. Clear differences were observed between these groups and the group that received only Gem (2.7-fold increase, *p* < 0.1584). The increase in the expression of *GADD45α* for the radiation treatment groups was also greater than the group that received Gem alone. The expression of *BRCA1* was significantly down-regulated after treatment with Gem/^212^Pb-trastuzumab (-3.2-fold decrease, *p* < 0.002) and Gem/^212^Pb-HuIgG (-3.1 fold decrease, *p* < 0.0028) compared to the group that received Gem alone (-1.1-fold decrease, *p* < 0.6248) (Table 1).

**Table 1:** Differential expression of genes involved in apoptosis in LS-174T i.p. xenografts following treatment with Gemcitabine and α-treatment.

Symbol	Gene name	GeneBank ID	Fold Change					
			Gemcitabine-^212^Pb-trastuzumab	*p*	Gemcitabine-^212^Pb-HuIgG	*p*	Gemcitabine	*p*
*BRCA1*	Breast Cancer 1, early onset	NM007294	-3.2	0.0020	-3.1	0.0028	-1.1	0.6268
*CIDEA*	Cell death-inducing DEFA-like effector a	NM001279	2.7	0.1447	3.9	0.0145	3.0	0.0001
*GADD45α*	Growth arrest and DNA-damage-inducible, alpha	NM001924	4.5	0.0047	5.8	0.0004	3.0	0.0005
*GADD45γ*	Growth arrest and DNA-damage-inducible, gamma	NM006705	5.0	0.0001	5.9	0.0003	6.2	0.0143
*IP6K3*	Inositol hexakisphosphate kinase 3	NM054111	2.1	0.0241	1.2	0.4338	2.8	0.0005
*PCBP4*	Poly(rC)binding protein 2	NM020418	2.7	0.0048	3.2	0.0001	3.0	0.0011
*RAD21*	RAD21 homolog	NM006265	-2.4	0.0003	-2.2	0.0002	-2.2	0.0012
*p73*	Tumor protein p73	NM005427	8.6	0.0021	10.1	0.0005	2.7	0.1584

Mice bearing i.p. LS-174T xenografts were treated by Gem/^212^Pb-trastuzumab for 24h. qRT-PCR array was used for gene expression analysis in three independent experiments. The numbers indicate fold change compared to untreated control (2-fold change cut-off). Additional groups included gemcitabine alone and Gem/^212^Pb-HuIgG as a nonspecific control antibody. Results represent the average of a minimum of three replicates. A *p*-value < 0.05 was considered significantly significant.

### Gem/α-radiation treatment-induced tumor cytotoxicity may be associated with differentially expression of genes in the regulation of cell cycle arrest and cell cycle check point

The panel of genes in this study contained 15 cell cycle arrest and 8 cell cycle checkpoint regulatory genes. Of the 23 genes in these two categories, 6 genes (*CHK1*, *CHK2*, *GTSE1*, *BRCA1*, *FANCG*, and *NBN*) showed a >2 fold decrease and 4 genes (*GADD45α*, *MAP2K6*, *PCBP4*, and *SESN*) showed a >2 fold increase in expression from Gem/^212^Pb-trastuzumab treatment (Table 2). For tumors treated with Gem/^212^Pb-HuIgG, four genes (*CHK1*, *GTSE1*, *BRCA1*, and *FANCG*) decreased >2 fold while another 4 genes (*GADD45α*, *MAP2K6*, *PCBP4*, and *SESN*) showed a > 2 fold increase in expression. For those that decreased in expression, the level of fold change tended to be greater following the Gem/^212^Pb-trastuzumab treatment than from Gem/^212^Pb-HuIgG treatment. The inverse effect was exhibited for the genes whose expression increased whereby Gem/^212^Pb-HuIgG treatment tended to result in an enhanced level of effect versus that from Gem/^212^Pb-trastuzumab treatment. The greatest difference in the expression of *CHK1* and *GTSE1* was associated with Gem/^212^Pb-trastuzumab treatment versus tumors that received Gem/^212^Pb-HuIgG treatment. Additionally, five genes showed a change in expression that was > 2 fold due to treatment with Gem alone. With the exception of *NBN*, the level of expression tended to be lower than both the Gem/^212^Pb-trastuzumab and Gem/^212^Pb-HuIgG treatments.

**Table 2:** Differential expression of genes involved in cell cycle in LS-174T i.p. xenografts by Gemcitabine and α-treatment.

Symbol	Gene name	GeneBank ID	Fold Change					
			Gemcitabine-^212^Pb-trastuzumab	*p*	Gemcitabine-^212^Pb-HuIgG	*p*	Gemcitabine	*p*
*BRCA1*	Breast Cancer 1, early onset	NM007294	-3.2	0.0020	-3.1	0.0028	-1.1	0.6268
*CHK1*	CHK1 checkpoint homolog	NM001274	-4.5	0.0001	-3.4	0.0002	-1.6	0.0029
*CHK2*	CHK2 checkpoint homolog	NM007194	-2.6	0.0015	-1.9	0.0051	-1.0	0.8729
*FANCG*	Francomianemia, complementation group G	NM004629	-2.8	0.0007	-2.1	0.0019	1.5	0.0788
*GADD45α*	Growth arrest and DNA-damage-inducible, alpha	NM001924	4.5	0.0047	5.8	0.0004	3.0	0.0005
*GTSE1*	G-2 and S-phase expressed 1	NM016426	-5.2	0.0020	-4.3	0.0025	-2.0	0.0152
*MAP2K6*	Mitogen activated protein kinase kinase 6	NM002758	2.1	0.0032	-2.4	0.0047	1.7	0.0078
*NBN*	Nibrin	NM002485	-2.1	0.0068	-1.8	0.0093	-3.0	0.0032
*PCBP4*	Poly(rC)binding protein 2	NM020418	2.7	0.0048	3.2	0.0001	3.0	0.0011
*SESN1*	Sestrin1	NM014454	3.7	0.0011	3.9	0.0023	2.9	0.0181

Four genes associated with cell cycle arrest (*GADD45α*, *MAP2K6*, *PCBP4*, and *SESN1*) demonstrated increased expression while three genes (*CHK1*, *CHK2*, and *GTSE1*) decreased in expression (Table 2). Three genes (*BRCA1*, *FANCG*, and *NBN*) associated with cell cycle checkpoint elicited a decrease in gene expression. Of those genes, an alteration in *BRCA1* (-3.2-fold decrease, *p* < 0.0020) and *FANCG* (-2.8-fold decrease, *p* < 0.0007) gene expression was noted when compared to those tumors that were treated with Gem (Gem/^212^Pb-trastuzumab *vs* Gem, *p* < 0.05). In contrast, no significant differences in gene expression were observed for those same genes for Gem/^212^Pb-trastuzumab versus Gem/^212^Pb-HuIgG treated tumors.

### α-Radiation plus gemcitabine-induced cell killing is associated with a decrease in expression of damaged DNA repair genes

The profiling study using the PCR array also demonstrated that several genes associated with DNA repair pathways were significantly affected after exposure to GEM/^212^Pb-trastuzumab (Table 3). Genes pivotal in major DNA repair pathways including nucleotide excision repair (NER), base-excision (BER), mismatch repair (MMR), and double-strand break repair (DSB) are categorized in S1 Table. A total of twelve genes (*BRCA1*, *DMC1*, *EXO1*, *FANCG*, *FEN1*, *MSH2*, *PRKDC*, *RAD18*, *RAD51B*, *p73*, *UNG*, and *XRCC2*) were found to be clearly impacted in those tumors treated with Gem/^212^Pb-trastuzumab versus those treated with Gem alone. Interestingly, only three genes (*RAD18*, *XRCC2*, and *p73*) among these twelve demonstrated a significant difference between the tumors treated with Gem/^212^Pb-trastuzumab and Gem/^212^Pb-HuIgG. *RAD18* and *XRCC2* fall into a category of genes related to damaged DNA binding (DDB) while *p73* is involved in MMR. As noted previously for *p73*, tumors from the Gem/^212^Pb-HuIgG group demonstrated a somewhat higher increase (Gem/^212^Pb-trastuzumab *vs*. Gem/^212^Pb-HuIgG, *p* < 0.05) than those tumors treated with Gem/^212^Pb-trastuzumab (a 8.6-fold increase, *p* < 0.00219 *vs*. a 10.7-fold increase, *p* < 0.0005). *RAD18* and *XRCC2* demonstrated a decrease (-3.2-fold decrease, *p* < 0.0001; -3.2-fold decrease, *p* < 0.0006) in expression as well as a significant difference between Gem/^212^Pb-trastuzumab and Gem/^212^Pb-HuIgG, (*p* < 0.05) treated tumors. The rest of the genes among these twelve were all down-regulated. However, the differences in gene expression for those genes between the Gem/^212^Pb-trastuzumab and Gem/^212^Pb-HuIgG treated tumor tissue were negligible. Seven genes (*BRCA1*, *DMC1*, *FANCG*, *FEN1*, *MSH2*, *RAD18*, and *RAD51B*) are involved in DDB while *EXO1* and *MSH2* are associated with MMR. *FEN1* and *PRKDC* are involved in DSB repair while *UNG* is the only gene related to BER.

**Table 3:** Differential expression of gene expression involved in DNA repair in LS-174T i.p. xenografts by Gemcitabine and α-treatment.

Symbol	Gene name	GeneBank ID	Fold Change					
			Gemcitabine-^212^Pb-trastuzumab	*p*	Gemcitabine-^212^Pb-HuIgG	*p*	Gemcitabine	*p*
*BRCA1*	Breast Cancer 1, early onset	NM007294	-3.2	0.0020	-3.1	0.0028	-1.1	0.6268
*BTG2*	BTG family, member 2	NM006763	4.6	0.0001	4.9	0.0004	4.8	0.0001
*DMC1*	DNC1 dose suppressor of mck1 homolog	NM007068	-3.0	0.0009	-2.9	0.0046	-1.2	0.0519
*ERCC1*	Excision repair cross-complementing rodent repair efficiency, complementation group1	NM001983	2.6	0.0289	3.0	0.0001	1.8	0.0436
*EXO1*	Exonuclease 1	NM130398	-3.9	0.0003	-3.2	0.0003	-2.1	0.0030
*FANCG*	Fanconi anemia, complementation group G	NM004629	-2.8	0.0007	-2.1	0.0019	1.5	0.0788
*FEN1*	Flap structure-specific endonuclease 1	NM004111	-3.1	0.0035	-2.9	0.0039	-1.3	0.1582
*MSH2*	MutS homolog 2	NM000251	-3.3	0.0059	-3.0	0.0068	-2.1	0.0151
*MSH3*	MutS homolog 3	NM002439	-2.0	0.0752	-1.8	0.0898	-1.8	0.0113
*NBN*	Nibrin	NM002485	-2.1	0.0068	-1.8	0.0093	-3.0	0.0032
*NTHL1*	Nth endonuclease III-like 1	NM002528	-2.1	0.0006	-1.8	0.0001	-1.9	0.0002
*OGG1*	8-oxoguanine DNA glycosylase	NM002542	-2.0	0.1458	-1.9	0.1657	-1.5	0.2915
*PRKDC*	Protein kinase, DNA-activated, catalytic polypeptide	NM006904	-3.0	0.0100	-2.6	0.0112	-2.0	0.0204
*RAD18*	RAD18 homolog	NM020165	-3.2	0.0001	-2.2	0.0001	-1.0	0.9928
*RAD21*	RAD21 homolog	NM006265	-2.4	0.0003	-2.2	0.0002	-2.2	0.0012
*RAD51B*	RAD51 homolog B	NM133509	-2.4	0.0072	-1.9	0.0098	-1.1	0.4491
*SEMA4A*	Semadomain, immunoglobulin domain, cycloplastic domain 4A	NM022367	2.2	0.0795	2.9	0.0030	3.7	0.0002
*p73*	Tumor protein p73	NM005427	8.6	0.0021	10.7	0.0005	2.7	0.1584
*UNG*	Uracil-DNA glycosylase	NM003362	-2.9	0.0010	-2.2	0.0017	-1.2	0.0450
*XPC*	Xeroderma pigmentosum, complementation group C	NM004628	4.9	0.0004	5.1	0.0003	5.7	0.0211
*XRCC2*	X-ray repair complementing defective repair in Chinese hamster cells 2	NM005431	-3.2	0.0006	-2.2	0.0007	-1.2	0.2123

### ^212^Pb-trastuzumab with gemcitabine pre-treatment may interfere with DNA damage repair

**Fig 1:**
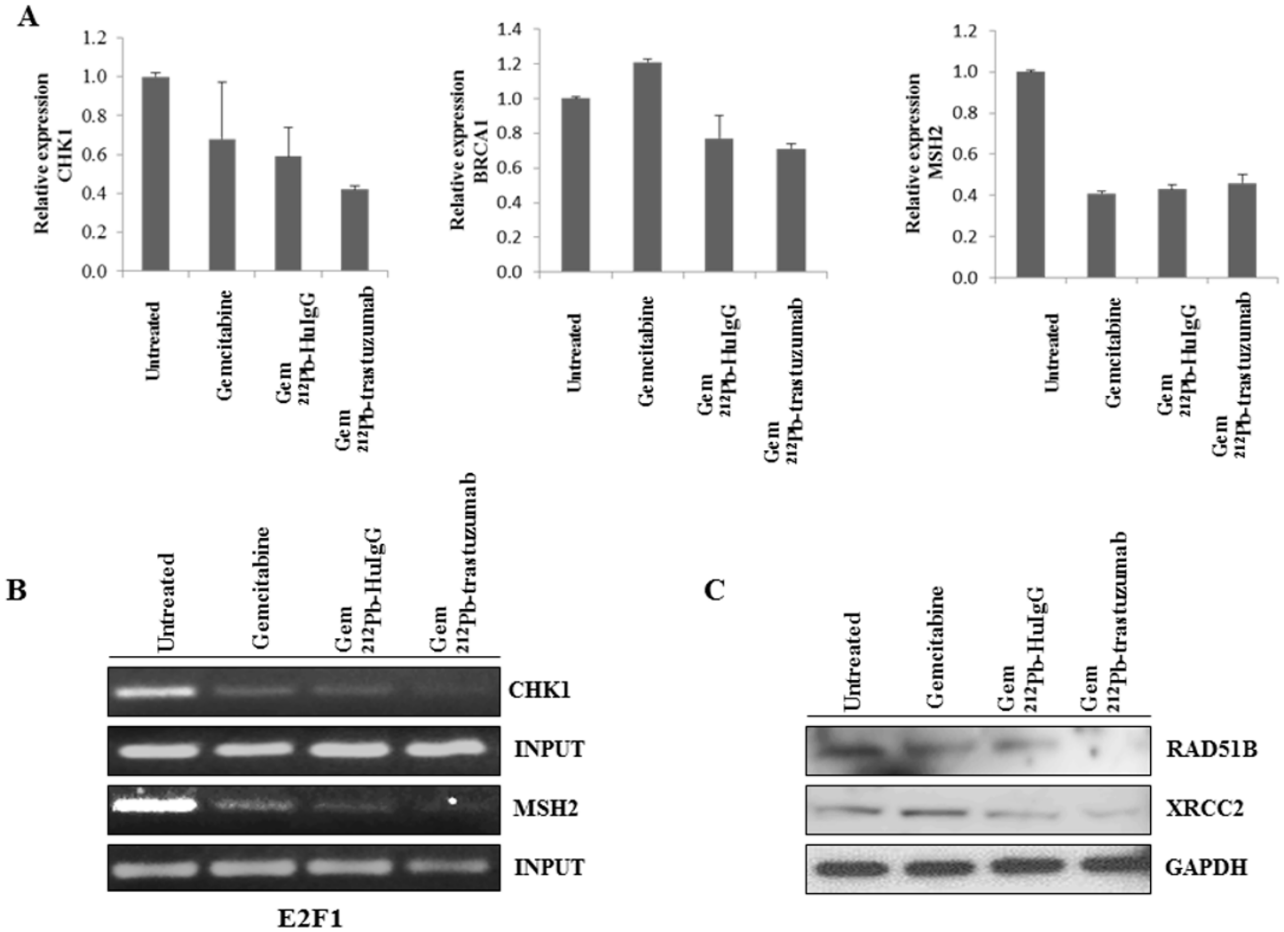
Expression of genes related to BASC (BRCA1-associated genome surveillance complex) and HRR in response to sequential treatment with Gem and ^212^Pb-trastuzumab. Mice bearing i.p. LS-174T xenografts were pre-treated with Gem followed 24 h thereafter with ^212^Pb-RIT. (A) Expression of *BRCA1*, *CHK1*, *MSH2* and was determined by qRT-PCR. Results represent the average of a minimum of three replications. (B) Binding abundance to E2F1 was determined by ChIP using specific primers for *CHK1* and *MSH2*. (C) Immunoblot analysis for RAD51B and XRCC2 was performed with tumor tissue collected as described. RAD51B and XRCC2 were detected at 32 kDa and 42 kDa, respectively. Equal protein loading control was GAPDH.

Based on the differentially expressed genes, further inquiry into possible pathways involved in the cell killing effect of Gem/^212^Pb-trastuzumab was initiated. Among those genes identified in the gene expression profile, *BRCA1*, *MSH2*, *MSH3*, and *NBN* were found down-regulated after exposure to α-radiation with Gem pretreatment. These genes are involved in *BRCA1*-associated genome surveillance complex (BASC) complex composed of *MSH2*, *MSH3*, *MSH6 and MLH1*, as well as *ATM*, *NBN*, *MRE11*, and *BLM* [28]. The expression of *BRCA1* and *MSH2* was determined at the transcriptional level to investigate the effect of targeted α-radiation on BASC. In response to Gem/^212^Pb-trastuzumab and Gem/^212^Pb-HuIgG treatment, expression of *BRCA1* at the transcriptional level was attenuated to a greater degree than the treatment of Gem only suggesting that defects in transcription-coupled repair systems including mismatch repair (*MSH2*) and DNA double stand repair (*BRCA1*) might occur (Fig 1A).

A greater reduction in the expression of *CHK1* is also evident in the LS-174T tumors that had been treated with Gem/^212^Pb-trastuzumab (*p* < 0.05) and Gem/^212^Pb-HuIgG (*p* < 0.05). *CHK1* and *MSH2* have binding sites for E2F, a transcription factor which is involved in DNA replication and DNA damage repairs [29, 30]. To investigate whether E2F may mediate an expression of those genes by recruitment of E2Fs to their promoter regions following the combined treatment of GEM/^212^Pb-trastuzumab, the binding of E2F1 to the *CHK1* and *MSH2* promoters were evaluated using a ChIP assay. As shown in Fig 1B, the association of E2F1 on *CHK1* and *MSH2* promoters appeared to be attenuated by Gem/^212^Pb-trastuzumab and Gem/^212^Pb-HuIgG treatment, suggesting that modulation of these genes may occur via a decrease in binding of the active transcription factor, E2F1, to the promoter region.

Among genes identified in the gene expression profile, *XRCC2* and *RAD51B*, which are RAD51 paralogs [31], appeared to be down-regulated after exposure to Gem/^212^Pb-trastuzumab. To examine the effect of Gem/^212^Pb-trastuzumab on damaged DNA repair, the expression of RAD51B and XRCC at the protein level were determined using immunoblot analysis. The results indicated that Gem/^212^Pb-trastuzumab attenuated expression in both proteins, suggesting the inefficient HR repair by Gem/^212^Pb-trastuzumab may be involved (Fig 1C).

### Cell killing induced by Gem/α-radiation treatment may be associated with p73 signaling

**Fig 2:**
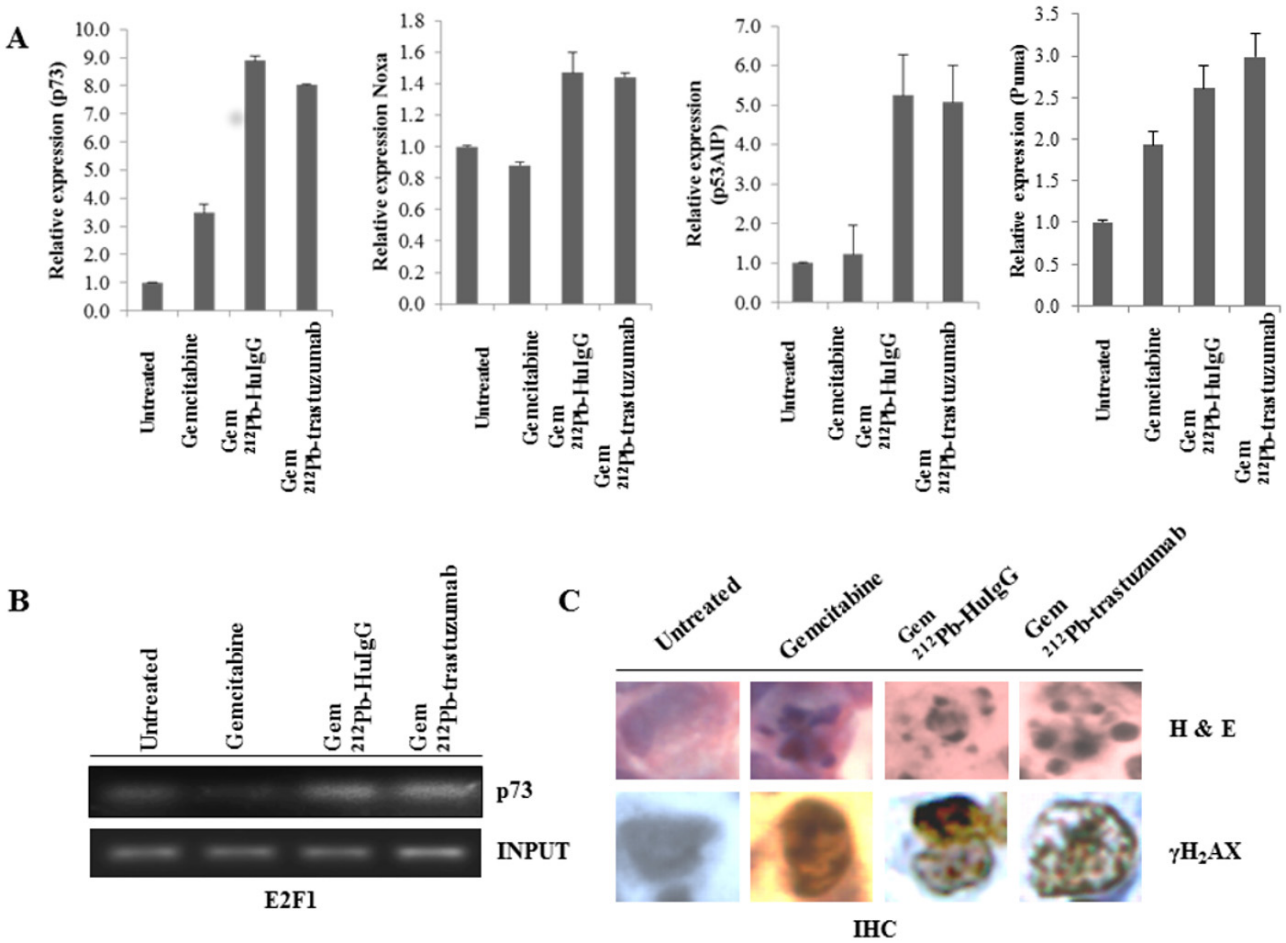
Gem/^212^Pb-trastuzumab may induce expression of *p73*, resulting in apoptosis. Mice bearing i.p. LS-174T xenografts were pre-treated with Gem followed 24 h thereafter with ^212^Pb-RIT. (A) Expression of *p73*, *NOXA*, *p53AIP1*, *and PUMA* was determined by qRT-PCR. Results represent the average of a minimum of three replications. (B) Binding abundance to E2F1 was determined by ChIP using specific primer for *p73*. (C) Immunohistochemical analysis using γH_2_AX and H&E staining was performed with tumor tissue collected as described.

Gem/^212^Pb-trastuzumab treatment significantly altered the expression of *p73* (8.6-fold increase, *p* < 0.0021) as demonstrated in the gene profiling study. To investigate the role of *p73* induced apoptosis in LS-174T i.p. xenografts harvested from mice treated with Gem/^212^Pb-trastuzumab and Gem/^212^Pb-HuIgG, the expression of *p73* at the transcriptional level was first determined using PCR. Expression of *p73* was significantly increased in the tumors treated with Gem/^212^Pb-trastuzumab as compared the ones treated with Gem only (Gem/^212^Pb-trastuzumab *vs*. Gem, *p* < 0.05). Expression of down-stream effectors of *p73* including *NOXA*, *PUMA*, and *P53AIP1* was also examined at the transcription level (Fig 2A). Gem/^212^Pb-trastuzumab increased the expression of *NOXA*, *PUMA*, and *P53AIP1*, compared to Gem only treated tumor (Gem/^212^Pb-trastuzumab *vs*. Gem, *NOXA* and *PUMA*, *p* < 0.05; *P53AIP1*, *p <* 0.01). There were only modest to negligible differences between Gem/^212^Pb-trastuzumab and Gem/^212^Pb-HuIgG treated tumors amongst these genes. *p73* is also an *E2F* target gene [32]. ChIP analysis revealed abundant *E2F1* on the *p73* promoter to effect increased expression in both the Gem/^212^Pb-trastuzumab and Gem/^212^Pb-HuIgG treatment groups, suggesting the *E2F1/p73* signaling may be activated after exposure to α-radiation with Gem pretreatment (Fig 2B). Next, to determine whether Gem/^212^Pb-trastuzumab induces DNA damage and apoptosis, immunohistochemistry (IHC) was performed using γH_2_AX and Haemotoxylin and Eosin (H&E) staining. DNA double strand damage and multi-micronuclei was evident from α-radiation with Gem pretreatment at 24 h as compared to the control groups (Fig 2C), indicating that DNA damage by α-radiation with ^212^Pb potentiates cell death to a greater extent than a Gem mono-therapy.

## Discussion

There is no guarantee that conventional radiation therapy procedures will consistently result in an efficient therapeutic response for the treatment of undetected metastatic or disseminated cancers. Targeted α-radiation therapy using biological vectors such as monoclonal antibodies (mAbs) against tumor associated antigens, may serve as magic bullets in a coordinated strategy to cure these diseases [9]. Targeted α-particle therapy with ^212^Pb-trastuzumab was successfully applied for the treatment of disseminated i.p. disease in murine xenograft models [14–16]. Based on these preclinical results, clinical translation to a Phase I trial has been successfully performed without toxicity at the University of Alabama [33, 34]. Gemcitabine is a clinically proven radiation sensitizer and improves therapeutic response in the treatment of locally advanced, metastatic and non-metastatic diseases [17]. Therapeutic efficacy of ^212^Pb-trastuzumab was even greater when employed with addition of Gem to the treatment protocol in the LS-174T i.p. tumor xenograft model [15]. Yong et al recently demonstrated that application of the combined modality of Gem/^212^Pb-trastuzumab not only abrogated G2 arrest but also impaired DNA damage repair in the same model [35]. To further understand *in vivo* mechanisms on a molecular basis, gene expression profiling was performed in LS-174T i.p. tumor xenografts after exposure to ^212^Pb-trastuzumab and gemcitabine.

Herein, a total of 84 genes associated with DNA damage response were analyzed using a real-time quantitative PCR (qRT-PCR) array 24 h after Gem/^212^Pb-RIT treatment of LS-174T tumor xenografts. In each of the functionally classified categories such as apoptosis, cell cycle regulation, and damaged DNA repair (S1 Table), differentially expressed genes by Gem/^212^Pb-trastuzumab were compared to Gem mono-therapy. In many of these instances the level of gene expression was similar to Gem/^212^Pb-HuIgG, an indication that a strong α-radiation effect occurs in the presence of Gem.

Six genes (*CIDEA*, *GADD45α*, *GADD45γ*, *IP6K3*, *PCBP4*, and *p73*) involved in apoptosis were affected in the α-particle radiation treatment groups. Increased expression following α-particle radiation treatment was greater for *p73* and *GADD45α* than for the group that received just Gem. In response to DNA damage, *p73/GADD45* has been known to induce cell cycle arrest and cell death. Indeed, the induction of G2/M arrest and apoptosis through the *p73/GADD45* signaling pathway by ^212^Pb-trastuzumab treatment has been recently reported from Yong et al [36].

Ten genes involved in the cell cycle were differentially regulated by Gem/^212^Pb-trastuzumab compared to Gem mono-therapy. The effect of Gem alone was not pronounced in those genes. *BRCA1*, *CHK1*, *CHK2*, *GTSE1*, and *FANCG* were down-regulated by Gem/^212^Pb-trastuzumab treatment. While alteration in gene expression between Gem/^212^Pb-trastuzumab and Gem/^212^Pb-HuIgG was negligible for some of the genes, expression of *CHK1* (-4.5-fold, *p* < 0.0001) after Gem/^212^Pb-trastuzumab treatment was substantially lower compared to either the Gem/^212^Pb-HuIgG or Gem treatments. Sensitization of tumor cells to cell death through inhibition of the DNA damage response is a promising strategy for enhancing therapeutic efficacy in the treatment of cancers. As a mediator of DNA damage response, checkpoint kinase 1 (*CHK1*) generally coordinates cell cycle arrest and DNA damage repair. *CHK1* and *CHK2* have been found to play pivotal roles in checkpoint functions of *ATR* and *ATM*. In fact, *CHK1* deficiency has been found to inhibit the activation of G2/M resulting in suppression of proliferation in response to radiation [37–39]. Thus, decreased *CHK1* and *CHK2* expression by a combined Gem/^212^Pb-trastuzumab treatment may be significant to the response of the cancer cells.

Among those genes associated with DNA repair, twelve genes (*BRCA1*, *DMC1*, *EXO1*, *FANCG*, *FEN1*, *MSH2*, *PRKDC*, *RAD18*, *RAD51B*, *p73*, *UNG*, and *XRCC2*) were differentially expressed in the LS-174T tumor xenografts following Gem/^212^Pb-trastuzumab and Gem/^212^Pb-HuIgG treatments. Gem mono-therapy resulted in negligible effects on these twelve genes in this study. Compared to results in a previous study that related treatment with ^212^Pb-trastuzumab alone [36], more genes were down-regulated in their expression by the Gem/^212^Pb-trastuzumab and Gem/^212^Pb-HuIgG, suggesting compromised efforts to overcome the stressful conditions invoked by a combined modality of targeted α-radiation and Gem. Comparison of the differential expression of the DNA damage repair genes shows a greater negative expression for *XRCC2* and *RAD18* for the Gem/^212^Pb-trastuzumab group than the Gem/^212^Pb-HuIgG group. However, for most of the other genes, the difference in the gene expression response between the two groups was negligible.

Among those genes identified in the profile, the four involved in the BRCA1-associated genome surveillance complex (BASC), *BRCA1*, *MSH2*, *MSH3*, and *NBN*, were down-regulated by Gem/^212^Pb-trastuzumab and Gem/^212^Pb-HuIgG. In response to DNA damage, BASC may play an important role as sensors of abnormal DNA structure or as effectors of DNA damage repair [28]. Loss of *BRCA1* function results in abnormal G2/M checkpoint, causing genetic instability [40, 41]. The defect in *MSH2* function is associated with inhibition of *CHK1* and *CHK2* and abrogated *RAD51*, leading to suppression of cell proliferation in response to radiation [42, 43]. As indicated in the results, aberrant regulation of the BRCA1-associated target genes such as *CHK1* and *MSH2* was observed by ChIP analysis. The observed results here suggest that a defective ATR/CHK1 signaling pathway mediated by *BRCA1/MSH2* may be involved in suppression of cell proliferation by Gem/^212^Pb-trastuzumab. Interaction of *CHK1* and *RAD51*, which are required for HR, may be disrupted in *BRCA1* deficient cells [44]. Defects in the *RAD51* paralog genes result in abnormal recombinational repair, causing genomic instability [31]. Treatment with Gem/^212^Pb-trastuzumab also down-regulated expression of *RAD51B*, and *XRCC2* as evidenced by the gene expression profiling and immunoblot analysis. These observations suggest that maintenance of genomic integrity through recombinational repair may be impaired by Gem/^212^Pb-trastuzumab. Previously, sensitization of tumor treated with Gem/^212^Pb-trastuzumab was shown to result in inhibition of checkpoint and impaired DNA damage repair [35]. As observed here, the lower expression of *CHK1*, *MSH2*, *BRCA1*, and *RAD51* palalog genes together bolsters the earlier findings. The failure to correctly perform checkpoint response and DNA repair could also correlate with the observed inability to maintain the G2/M arrest by Gem/^212^Pb-trastuzumab. Therefore, targeting genes associated with the checkpoint signaling pathway and also DNA damage repair may be an attractive therapeutic strategy to take advantage of these two interlinked processes.

*p73* is functionally and structurally related to *p53* [45]. The α-particle radiation and Gem combined modality effects a greater cell killing more than likely through activation of the *p73* signaling pathway as previously observed when tumors were treated with just ^212^Pb-trastuzumab [36]. Indeed, up-regulation of *p73* also induced expression of its downstream effectors (*NOXA*, *PUMA*, and *p53AIP1*) by Gem/^212^Pb-trastuzumab, suggesting that the *GADD45/p73* signaling pathway is activated after exposure to the combination of Gem and ^212^Pb-trastuzumab. ChIP analysis elicited an increased binding capacity of E2F1 on the *p73* promoter, suggesting that the enhancement of apoptosis may be associated with active E2F1/p73 signaling. In *p53* inactivated cells, up-regulation of *p73* expression is mediated through E2F-1, suggesting an intrinsic rescuing mechanism may occur to compromise the loss of p53 function [46]. *In vivo* cell death mechanisms by the α-particle radiation are tremendously complex in the interlinked biological processes. Among those genes modulated after exposure to α-particle radiation, *GADD45*, *IP6K3* (inositol hexakisphosphate kinase 3), and *PCBP4* (Poly(rC)-binding protein 4) have been previously known to be mediated by *p53*-regulated signaling pathway, leading to apoptosis. However, gene expression of *p53* has not been observed in gene profiling after either exposure to ^212^Pb-TCMC-trastuzumab [36] or Gem/^212^Pb-TCMC-trastuzumab. It has been known that RIT may induce lethal impact on radio-resistant tumors regardless of *p53* gene status. Therefore, activation of *p73* may play a pivotal role in the interlinked biological processes, leading to cell death in tumors that lack a *p53*-regulated signaling pathway.

**Fig 3:**
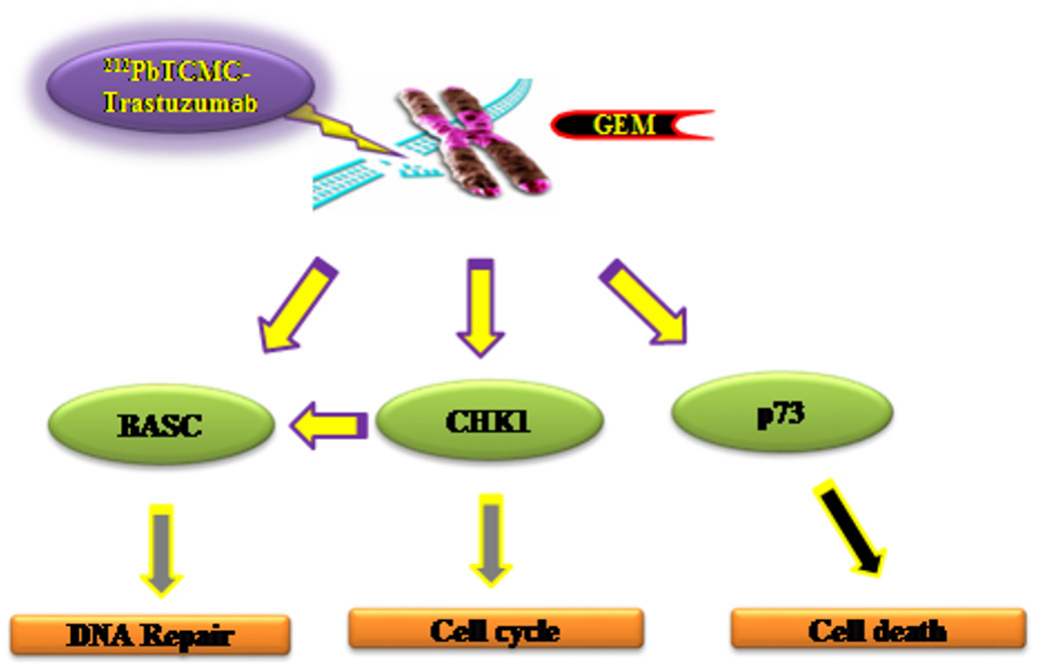
Interplay between DNA damage repair and check point signaling in the stressful growth arrest conditions by Gem/ ^212^Pb-trastuzumab. See text for details.

The possibly predicted pathways that control cell cycle arrest and DNA damage repair, resulting in cell death have been demonstrated as depicted in Fig 3. DNA repair and checkpoint response are two interlinked processes. In response to the combined treatment of Gem and ^212^Pb-trastuzumab, one must note that there is an extensive interplay between the signaling pathways of checkpoint and DNA damage repair leading to severe growth arrest. While a need to improve the therapeutic efficacy of α-particle RIT combined with chemotherapy exists, the successful development and application of new tools such as a gene expression profiling and the elucidation of the fundamental molecular mechanisms in action during these combination therapies could aid in optimization of the combinations of chemotherapy reagents with radiation therapy as well as the sequence of their administration leading to augmented and enhanced radiotherapy choices for future clinical trials.

## Supporting Information

S1 Table Functional gene grouping. Comparison of the relative expression of 84 DNA damage related genes involved in apoptosis (Table 1), cell cycle (Table 2), and DNA damage repair (Table 3) was characterized with the human DNA damage signaling pathway PCR array.
(PPT)
