# Supplementary material for: Cell Killing Mechanisms and Impact on Gene Expression by Gemcitabine and 212Pb-Trastuzumab Treatment in a Disseminated i.p. Tumor Model
Source: PLoS One. 2016 Jul 28;11(7):e0159904. doi: 10.1371/journal.pone.0159904 (PMC4965152; doi:10.1371/journal.pone.0159904)
Supplement: S1 Table — Comparison of the relative expression of 84 DNA damage related genes involved in apoptosis (Table 1), cell cycle (Table 2), and DNA damage repair (Table 3) was characterized with the human DNA damage signaling pathway PCR array. (PPT) [file pone.0159904.s001.ppt]

## Slide 1
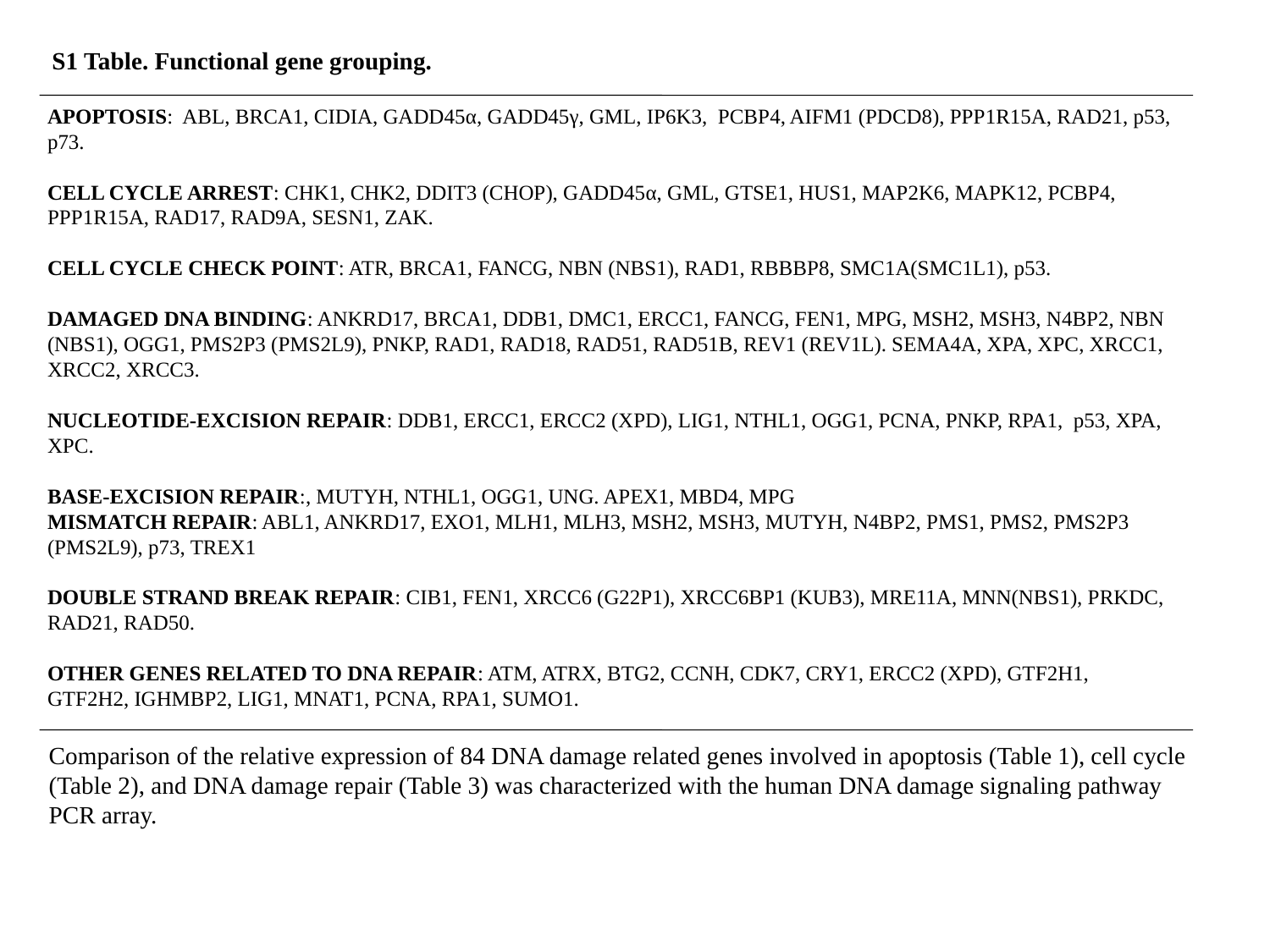

S1 Table. Functional gene grouping.
APOPTOSIS: ABL, BRCA1, CIDIA, GADD45α, GADD45γ, GML, IP6K3, PCBP4, AIFM1 (PDCD8), PPP1R15A, RAD21, p53, p73.
CELL CYCLE ARREST: CHK1, CHK2, DDIT3 (CHOP), GADD45α, GML, GTSE1, HUS1, MAP2K6, MAPK12, PCBP4, PPP1R15A, RAD17, RAD9A, SESN1, ZAK.
CELL CYCLE CHECK POINT: ATR, BRCA1, FANCG, NBN (NBS1), RAD1, RBBBP8, SMC1A(SMC1L1), p53.
DAMAGED DNA BINDING: ANKRD17, BRCA1, DDB1, DMC1, ERCC1, FANCG, FEN1, MPG, MSH2, MSH3, N4BP2, NBN (NBS1), OGG1, PMS2P3 (PMS2L9), PNKP, RAD1, RAD18, RAD51, RAD51B, REV1 (REV1L). SEMA4A, XPA, XPC, XRCC1, XRCC2, XRCC3.
NUCLEOTIDE-EXCISION REPAIR: DDB1, ERCC1, ERCC2 (XPD), LIG1, NTHL1, OGG1, PCNA, PNKP, RPA1, p53, XPA, XPC.
BASE-EXCISION REPAIR:, MUTYH, NTHL1, OGG1, UNG. APEX1, MBD4, MPG
MISMATCH REPAIR: ABL1, ANKRD17, EXO1, MLH1, MLH3, MSH2, MSH3, MUTYH, N4BP2, PMS1, PMS2, PMS2P3 (PMS2L9), p73, TREX1
DOUBLE STRAND BREAK REPAIR: CIB1, FEN1, XRCC6 (G22P1), XRCC6BP1 (KUB3), MRE11A, MNN(NBS1), PRKDC, RAD21, RAD50.
OTHER GENES RELATED TO DNA REPAIR: ATM, ATRX, BTG2, CCNH, CDK7, CRY1, ERCC2 (XPD), GTF2H1, GTF2H2, IGHMBP2, LIG1, MNAT1, PCNA, RPA1, SUMO1.
Comparison of the relative expression of 84 DNA damage related genes involved in apoptosis (Table 1), cell cycle
(Table 2), and DNA damage repair (Table 3) was characterized with the human DNA damage signaling pathway
PCR array.
